# Monkeypox Virus Infections After 2 Preexposure Doses of JYNNEOS Vaccine — United States, May 2022–May 2024

**DOI:** 10.15585/mmwr.mm7320a3

**Published:** 2024-05-23

**Authors:** Sarah Anne J. Guagliardo, Ian Kracalik, Rosalind J. Carter, Christopher Braden, Rebecca Free, Mukesh Hamal, Alexandra Tuttle, Andrea M. McCollum, Agam K. Rao

**Affiliations:** ^1^Mpox National Response Team, CDC; ^2^Division of High-Consequence Pathogens and Pathology, National Center for Emerging and Zoonotic Infectious Diseases, CDC.

**Summary**

**What is already known about this topic?**

Two JYNNEOS vaccine doses prevent mpox; however, infection in fully vaccinated persons can occur.

**What is added by this report?**

Monkeypox virus infection after receipt of 2 JYNNEOS doses is estimated to have occurred in <1% of fully vaccinated persons and comprises a small proportion of national cases. Among persons who experienced infection after having received a complete 2-dose series and for whom complete data were available, infections have been milder than those among unvaccinated persons. Disparate time intervals from vaccination to infection among fully vaccinated persons suggest that immunity is not waning.

**What are the implications for public health practice?**

To optimize protection, persons recommended to receive mpox vaccination should complete the 2-dose JYNNEOS vaccination series. No additional vaccine doses are recommended at this time.

## Abstract

Two doses of JYNNEOS vaccine are effective in preventing many mpox cases and can reduce the severity of symptoms in infected persons. However, infections among fully vaccinated persons can occur. During May 2022–May 2024, a total of 271 mpox cases among fully vaccinated persons were reported to CDC from 27 U.S. jurisdictions. These reported infections are estimated to have occurred in <1% of fully vaccinated persons. Compared with cases among unvaccinated persons, infections among fully vaccinated persons were more likely to occur among non-Hispanic White men aged 30–39 years, were associated with increased numbers of sexual partners, and resulted in less severe disease (p<0.001). Among infections in fully vaccinated persons with complete data, infections after vaccination were reported more commonly after receipt of heterologous (subcutaneous and intradermal) (46%) or homologous subcutaneous (32%) JYNNEOS vaccination than after homologous intradermal (22%) vaccination. Disparate time intervals from vaccination to infection among fully vaccinated persons suggest that immunity is not waning. The median interval between the second vaccine dose and illness onset was longer for cases among persons who had received 2 intradermal doses (median = 363 days; IQR = 221–444 days) compared with cases in persons who had received 2 subcutaneous doses (median = 263 days; IQR = 47–334 days) (p<0.001). The implications of this finding are not known; however, these data should increase confidence in the effectiveness of vaccine doses that were administered intradermally, the preferred method of administration during the peak of the outbreak when vaccine supply was limited. Persons recommended to receive the JYNNEOS vaccine should receive 2 doses, irrespective of the route of administration, and at this time, additional doses are not recommended for the affected population.

## Introduction

JYNNEOS is a replication-deficient orthopoxvirus vaccine approved as a 2-dose series for prevention of smallpox and mpox.* This product was administered to nearly all vaccinated U.S. persons with risk factors for mpox during the ongoing global outbreak, which has disproportionately affected gay, bisexual, and other men who have sex with men (MSM) and transgender and nonbinary persons^†^ (*1*). Real-world vaccine effectiveness studies have indicated that 2 JYNNEOS doses are effective in preventing many mpox cases (*2*–*4*); infections among persons who have received 2 JYNNEOS doses, when infection does occur, are less severe than are those among unvaccinated persons (*5*,*6*).

Most persons who received 2 JYNNEOS vaccine doses received both doses during 2022, but only 25% of the population at risk is estimated to have been fully vaccinated.^§^ Mpox case counts have decreased substantially in the United States since the peak of the outbreak; however, cases continue to occur, including, sometimes, among fully vaccinated persons (*7*). An mpox cluster recognized during May 2023 in Chicago, Illinois predominantly affected fully vaccinated persons (*7*) and raised several questions about the effectiveness of JYNNEOS vaccines, including the following: 1) the frequency of infection among fully vaccinated persons, 2) how often such infections are associated with receipt of 2 intradermal doses (i.e., the vaccination route less familiar to clinicians but preferentially recommended during the peak of the U.S. mpox response^¶^), 3) behavioral risk factors, and 4) potential need for booster doses. Public perception of an increase in monkeypox virus (MPXV) infections among fully vaccinated persons during 2024 has further fueled concerns about the 2-dose series. To answer these questions, including whether there are discernable indications for waning vaccine immunity, clinical and vaccination characteristics of nationally reported infections among fully vaccinated persons were evaluated.

## Methods

The Council of State and Territorial Epidemiologists recommends that U.S. health departments report probable and confirmed mpox cases** to CDC’s National Notifiable Diseases Surveillance System.^††^ Reported data include vaccination dates and demographic and clinical characteristics.^§§^ Probable or confirmed cases reported during May 11, 2022–May 1, 2024, were included in this analysis. An unvaccinated case was defined as a probable or confirmed mpox case in a person for whom 1) no history of vaccination with JYNNEOS was reported and 2) no vaccination dates were reported. Because a vaccine dose is considered to have maximum immunogenicity 14 days after administration, a case in a fully vaccinated person was defined as one in a person with documented receipt of 2 JYNNEOS doses ≥14 days before illness onset and with vaccination dates occurring since May 2022.^¶¶^ Cases among persons who had received a single vaccine dose or a second dose administered <14 days before mpox illness onset were excluded because these administration schedules are expected to provide suboptimal protection and are inconsistent with the recommended 2-dose series.

Cases among unvaccinated and fully vaccinated persons were compared by demographic characteristics, behaviors (i.e., number of recent sexual partners), and clinical presentations using chi-square and Fisher’s exact tests and odds ratios (ORs). Among fully vaccinated persons, the interval from receipt of the second vaccine dose to illness onset and the routes of vaccination (i.e., 2 subcutaneous doses [homologous subcutaneous], 1 subcutaneous and 1 intradermal dose [heterologous], and 2 intradermal doses [homologous intradermal]) were compared using a Kruskal-Wallis test. The interval from receipt of the second vaccine dose to illness onset was also compared for homologous subcutaneous and intradermal doses using the Wilcoxon rank-sum test. To determine how missing vaccination data might have skewed findings, demographic characteristics of mpox patients with complete data on vaccination status were compared with those of patients who were missing vaccination status using chi-square tests. To assess the frequency of reported MPXV infections among fully vaccinated persons, the proportion of such reported infections among the total number of fully vaccinated persons was estimated for a subset of 31 jurisdictions with vaccination status reported for 95% of persons with infection.*** The proportion of fully vaccinated cases among mpox cases included in this analysis was plotted over time. Analyses were conducted using R software (version 4.3.2; R Foundation). This activity was reviewed by CDC, deemed not research, and was conducted consistent with applicable federal law and CDC policy.^†††^

## Results

Among 32,819 probable or confirmed U.S. mpox cases reported to CDC during May 11, 2022–May 1, 2024, a total of 24,507 (75%) occurred in unvaccinated persons, and 271 (0.8%) occurred among persons who were fully vaccinated (Table 1); of these 271 cases, 51 (19%) occurred during 2024. Vaccination status was missing for 3,737 (11%) cases; an additional 4,304 (13%) cases were excluded from analysis because the patient had received only 1 JYNNEOS vaccine dose or had received the second dose <14 days before illness onset, or because other exclusion criteria were met. Cases among unvaccinated and fully vaccinated persons were reported from 54 and 27 U.S. jurisdictions, respectively. Approximately 80% of cases with missing vaccination status were reported from four jurisdictions. 

**TABLE 1:** Demographic and underlying clinical characteristics of persons with mpox, by JYNNEOS vaccination status — United States, May 2022–May 2024

Characteristic	No. (%)		p-value^†^	No. (%)		p-value^†^
	Fully vaccinated* n = 271	Unvaccinated n = 24,507		Total included (fully vaccinated and unvaccinated) N = 24,778	Missing vaccination status n = 3,737	
**Age group, yrs**						
<30	44 (16.5)	7,120 (29.1)	<0.001	**7,164 (28.9)**	921 (25.6)	<0.001
30–39	126 (47.4)	10,107 (41.2)		**10,233 (41.3)**	1,397 (38.8)	
40–49	61 (22.9)	4,869 (19.9)		**4,930 (19.9)**	796 (22.1)	
50–59	24 (9.0)	2,017 (8.2)		**2,041 (8.2)**	399 (11.1)	
≥60	11 (4.1)	392 (1.6)		**403 (1.6)**	88 (2.4)	
Unknown or missing	5	2		**7**	136	
**Race and ethnicity **						
AI/AN, non-Hispanic	0 (—)	92 (0.4)	<0.001^§^	**92 (0.4)**	16 (0.5)	<0.001
Asian, non-Hispanic	7 (2.7)	556 (2.4)		**563 (2.4)**	164 (5.2)	
Black or African American, non-Hispanic	37 (14.3)	8,149 (34.9)		**8,186 (34.6)**	664 (21.2)	
NH/PI, non-Hispanic	0 (—)	57 (0.2)		**57 (0.2)**	10 (0.3)	
White, non-Hispanic	153 (59.1)	6,509 (27.9)		**6,662 (28.2)**	1,002 (32.0)	
Hispanic or Latino	49 (18.9)	7,127 (30.5)		**7,176 (30.4)**	1,198 (38.3)	
Multiple races, non-Hispanic	7 (2.7)	341 (1.5)		**348 (1.5)**	21 (0.7)	
Other races, non-Hispanic	6 (2.3)	536 (2.3)		**542 (2.3)**	53 (1.7)	
Unknown or missing	12	1,140		**1,152**	609	
**Gender identity**						
Female	0 (—)	748 (3.6)	0.02^§^	**748 (3.5)**	79 (2.7)	<0.001^§^
Male	207 (99.0)	19,808 (94.5)		**20,015 (94.5)**	2,742 (94.4)	
Transgender female	0 (—)	136 (0.6)		**136 (0.6)**	9 (0.3)	
Transgender male	0 (—)	29 (0.1)		**29 (0.1)**	4 (0.1)	
Another gender or multiple genders	2 (1.0)	240 (1.1)		**242 (1.1)**	72 (2.5)	
Unknown or missing	62	3,546		**3,608**	831	
**HIV status **						
Persons with HIV	55 (29.3)	4,061 (53.7)	**<**0.001	**4,116 (53.2)**	215 (56.6)	0.2
Persons without HIV	133 (70.7)	3,495 (46.3)		**3,628 (46.8)**	165 (43.4)	
Unknown or missing	83	16,951		**17,034**	3,357	
**Immunocompromising condition (excluding HIV)**						
No	131 (91.0)	9,212 (85.3)	0.07	**9,343 (85.4)**	284 (78.0)	<0.001
Yes	13 (9.0)	1,583 (14.7)		**1,596 (14.6)**	80 (22.0)	
Unknown or missing	127	13,712		**13,839**	3,373	
**No. of recent sexual partners,^¶^ median (IQR) **	2 (1–3)	1 (1–2)	<0.001**	**1 (1–2)**	1 (1–3)	<0.001**
Unknown or missing	196	16,119		**16,315**	3,346	

**Abbreviations:** AI/AN = American Indian or Alaska Native; NH/PI = Native Hawaiian or Pacific Islander.

* Probable or confirmed cases in persons who received 2 JYNNEOS doses, with the most recent dose received ≥14 days before illness onset and with vaccination dates occurring since May 2022. ^†^ Chi-square test p-value except where noted; p<0.05 was considered statistically significant.  ^§^ Fisher’s exact test p-value.  ^¶^ During the 21 days before symptom onset.  ** Wilcoxon rank-sum test p-value.

Compared with unvaccinated cases, a higher proportion of fully vaccinated cases occurred among non-Hispanic White persons (59%) and persons aged 30–39 years (47%) (p<0.001, Fisher’s exact and chi-square tests, respectively). Information about the number of sexual partners was, over time, removed from the data requested by the CDC and, therefore, often missing, particularly during 2024. However, among 8,463 (34%) mpox patients with complete information on the number of sexual partners during the 21 days preceding symptom onset, the median number reported by those who were fully vaccinated (two; IQR = one to three) was higher than that reported by those who were unvaccinated (one; IQR = one to two) (p<0.001, Wilcoxon rank-sum test). A lower proportion of fully vaccinated patients were persons with HIV (29%) compared with unvaccinated patients (54%) (p<0.001, chi-square test). Compared with patients with complete data on vaccination status, missing data on vaccination status was more prevalent among mpox patients aged >40 years (36%), those of Hispanic or Latino ethnicity (38%), and those with an immunocompromising condition (excluding HIV) (22%). 

Mpox cases among fully vaccinated persons occurred a median of 266 days after receipt of the second JYNNEOS dose (range = 14–621 days; IQR = 64–420 days). The vaccine administration route^§§§^ of both doses was reported for 139 (51%) of 271 infected persons; among these persons, 64 (46%) received heterologous doses, 45 (32%) received homologous subcutaneous doses, and 30 (22%) received homologous intradermal doses. Whereas no significant difference was detected among all three groups, a difference was found between homologous vaccine recipients: among all homologous 2-dose recipients, the median interval from receipt of the second dose to illness onset among persons who had received 2 intradermal doses was 363 days (IQR = 221–444 days), and the median interval among those who had received 2 subcutaneous doses was 263 days (IQR = 47–334 days) (p<0.001). The impact of HIV infection on interval from vaccination to infection could not be assessed because of missing viral load and other data necessary to stratify cases by immunocompromised status. 

The odds of having systemic illness (e.g., fever, headache, lymphadenopathy, vomiting, abdominal pain, myalgia, chills, or malaise) were significantly lower among persons with mpox who were fully vaccinated than among those who were unvaccinated (p<0.05 for all tests) (Table 2). Although the median number of reported anatomic locations with rash was lower among fully vaccinated patients (one) than among unvaccinated patients (four), the odds of reported genital rash were higher among fully vaccinated patients than among those who were unvaccinated (OR = 2.3). Hospitalization was less prevalent among vaccinated persons with mpox (three [1.4%] of 212) than among unvaccinated persons (1,662 [8.4%] of 19,716) (OR = 0.2); a total of 56 deaths occurred among unvaccinated mpox patients, and none occurred among those who were fully vaccinated. 

**TABLE 2:** Clinical manifestations of persons with probable or confirmed mpox, by JYNNEOS vaccination status — United States, May 2022–May 2024

Characteristic	No. (%)		Odds ratio (95% CI)	p-value^†^
	Fully vaccinated* n = 271	Unvaccinated n = 24,507		
**Outcomes**				
**Hospitalized because of mpox**				
No	209 (98.6)	18,054 (91.6)	0.2 (0.0–0.5)	<0.001^§^
Yes	3 (1.4)	1,662 (8.4)		
Unknown or missing	59	4,791		
**Death due to mpox **				
No	116 (100.0)	13,521 (99.6)	Not tested	Not tested
Yes	0 (—)	56 (0.4)		
Unknown or missing	155	10,930		
**Systemic illness** ^¶^				
No	36 (28.3)	915 (12.3)	0.4 (0.2–0.5)	<0.001
Yes	91 (71.7)	6,514 (87.7)		
Unknown or missing	144	17,078		
**Fever**				
No	90 (61.6)	4,283 (36.6)	0.4 (0.3–0.5)	<0.001
Yes	56 (38.4)	7,412 (63.4)		
Unknown or missing	125	12,812		
**Headache **				
No	96 (66.2)	5,932 (47.5)	0.5 (0.3–0.7)	<0.001
Yes	49 (33.8)	6,559 (52.5)		
Unknown or missing	126	12,016		
**Lymphadenopathy **				
No	83 (57.2)	4,963 (47.1)	0.7 (0.5–0.9)	0.02
Yes	62 (42.8)	5,583 (52.9)		
Unknown or missing	126	13,961		
**Vomiting **				
No	124 (91.9)	7,313 (81.8)	0.4 (0.2–0.7)	0.004
Yes	11 (8.1)	1,631 (18.2)		
Unknown or missing	136	15,563		
**Abdominal pain **				
No	128 (94.1)	9,330 (86.2)	0.4 (0.2–0.8)	0.01
Yes	8 (5.9)	1,495 (13.8)		
Unknown or missing	135	13,682		
**Myalgia**				
No	98 (70.0)	5,129 (48.0)	0.4 (0.3–0.6)	<0.001
Yes	42 (30.0)	5,555 (52.0)		
Unknown or missing	131	13,823		
**Chills**				
No	93 (66.0)	5,456 (42.4)	0.4 (0.3–0.5)	<0.001
Yes	48 (34.0)	7,414 (57.6)		
Unknown or missing	130	11,637		
**Malaise**				
No	69 (47.6)	4,097 (38.0)	0.7 (0.5–0.9)	0.02
Yes	76 (52.4)	6,685 (62.0)		
Unknown or missing	126	13,725		
**Other symptoms**				
**No. of anatomic locations with rash, median (IQR) **	1 (1–2)	4 (2–6)	—	<0.001**
Unknown or missing	183	10,935		
**Genital rash **				
No	29 (33.0)	7,161 (52.8)	2.3 (1.4–3.7)	0.003
Yes	59 (67.0)	6,411 (47.2)		
Unknown or missing	183	10,935		
**Rash **				
No	9 (5.1)	375 (2.7)	0.5 (0.3–1.2)	0.09
Yes	168 (94.9)	13,293 (97.3)		
Unknown or missing	94	10,839	—	
**Rectal pain **				
No	90 (61.6)	6,489 (63.2)	1.1 (0.7–1.5)	0.8
Yes	56 (38.4)	3,780 (36.8)		
Unknown or missing	125	14,238		
**Proctitis **				
No	121 (91.0)	6,762 (85.1)	0.6 (0.3–1.0)	0.08
Yes	12 (9.0)	1,186 (14.9)		
Unknown or missing	138	16,559		
**Rectal bleeding **				
No	112 (80.0)	7,350 (79.1)	1.1 (0.7–1.7)	0.9
Yes	28 (20.0)	1,945 (20.9)		
Unknown or missing	131	15,212		
**Pus in stool **				
No	117 (84.2)	7,389 (82.4)	0.9 (0.5–1.4)	0.7
Yes	22 (15.8)	1,578 (17.6)		
Unknown or missing	132	15,540		
**Tenesmus**				
No	113 (81.9)	7,736 (82.3)	1.0 (0.6–1.6)	1.0^¶^
Yes	25 (18.1)	1,669 (17.7)		
Unknown or missing	133	15,102		
**Conjunctivitis**				
No	117 (95.9)	8,624 (95.5)	0.9 (0.3–2.2)	1.0
Yes	5 (4.1)	405 (4.5)		
Unknown or missing	149	15,478		

* Probable or confirmed cases in persons who received 2 JYNNEOS doses, with the most recent dose received ≥14 days before illness onset and with vaccination dates occurring since May 2022.

^† ^Chi-square test p-value except where noted; p<0.05 was considered statistically significant.

^§^ Fisher’s exact test p-value.

^¶^ Systemic illness includes the presence of fever, headache, lymphadenopathy, vomiting, abdominal pain, myalgia, chills, or malaise.

** Wilcoxon rank-sum test p-value.

Among 31 jurisdictions^¶¶¶^ with complete vaccination status for 95% of persons, 187 infections were reported among 188,907 fully vaccinated persons (with 2 JYNNEOS doses), resulting in a 0.1% infection rate. The number of breakthrough infections did not comprise a significant proportion of infections, including during 2024 (Figure).

**FIGURE:**
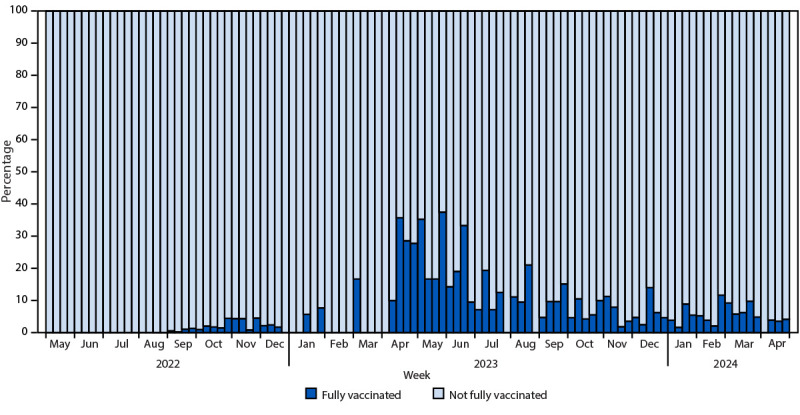
Proportion of fully vaccinated mpox cases* among all mpox cases, by epidemiologic week — United States, May 2022–May 2024 * Probable or confirmed cases in persons who received 2 JYNNEOS doses, with the most recent dose received ≥14 days before illness onset and with vaccination dates occurring since May 2022.

## Discussion

This report corroborates other published findings indicating that MPXV infection in fully vaccinated U.S. persons is rare and less severe regardless of the route of vaccination. For those for whom data on sexual exposures was reported, the difference between number of partners among fully vaccinated and unvaccinated cases was not large; however, similar to data from the May 2023 mpox cluster in Chicago, the results were statistically significant, suggesting that cases among fully vaccinated persons could be associated with increasing opportunities for MPXV exposure (*7*). Although no vaccine is 100% effective, persons who are fully vaccinated against mpox in the United States might have assumed that they are immune to infection and that other infection prevention strategies are no longer needed. Consistent with that, conclusions from the investigation in Chicago involving mpox cases in fully vaccinated persons were that they were likely due to frequent behaviors associated with mpox transmission, even with relatively high vaccine effectiveness and vaccine coverage (*7*). 

Despite a perceived increase in MPXV infections among fully vaccinated persons during 2024, this report indicates that, to date, persistent vaccine-derived immunologic response among persons who received the 2-dose vaccine series exists. Illness onset occurred during varied periods after the second vaccination dose, starting as early as 14 days and as late as 621 days after the second dose. Studies have indicated that vaccine titers decrease a few months after vaccination (*8*); this finding has spurred concerns that additional vaccine doses might be indicated. However, the clinical significance of waning antibody levels is uncertain. CDC laboratory data (PS Satheshkumar, CDC, unpublished data, 2024) and the findings from this report indicate that level of circulating titers is not the only marker of protection conferred by mpox vaccinations. The role of innate and cell-mediated immunity in preventing MPXV infections is not known, and the robustness of memory or recall response after an exposure might be more important determinants of disease outcome. 

With only one in four eligible U.S. persons fully vaccinated, clinicians and public health authorities should continue to focus efforts on increasing vaccine coverage, including among marginalized communities that are at risk for life-threatening mpox infections (*9*). During October 2023, the Advisory Committee on Immunization Practices (ACIP) recommended inclusion of JYNNEOS in the routine immunization schedule for persons at risk for mpox.**** Consistent with this recommendation, every opportunity should be taken to facilitate vaccination, including assessing behavioral risk factors and eligibility criteria during routine clinical appointments and vaccinating patients at those visits. Clinicians should remind patients that mpox is still circulating in the United States, and vaccination is an important tool in stopping the spread of mpox. In addition to vaccination, clinicians should educate patients about other prevention strategies such as talking with sex partners about any mpox signs and symptoms, being aware of any unexplained rashes or lesions on a partner’s body, and avoiding close or intimate contact if they or a sex partner become sick with mpox or experience an mpox-like rash. Mpox vaccination should be included as part of broader prevention activities and sexual health care, including HIV and other sexually transmitted infection (STI) testing and linkage to services, such as HIV preexposure prophylaxis or HIV treatment, as indicated.^††††^

### Limitations

The findings in this report are subject to at least three limitations. First, the total number of reported infections among fully vaccinated persons in this analysis might be underestimated because vaccination status was missing for 3,737 (11%) nationally reported cases; in addition, some mpox signs and symptoms after vaccination might be less severe (possibly subclinical), and therefore, might not have been evaluated by laboratory testing or included in national case counts. Second, the total number of cases among fully vaccinated persons was relatively small, which might have precluded detection of some associations, particularly for subgroup comparisons of interest (e.g., viral load status among patients with HIV). Finally, information about the type of sexual exposure was not reported and, therefore, could not be compared between vaccinated and unvaccinated cases. Relatedly, data about the number of sexual partners was reported for only a small number of patients because of changing reporting requirements over time. 

### Implications for Public Health Practice

CDC encourages clinicians and health departments to report vaccination status of persons with mpox because this data is essential to detecting waning immunity. When health departments identify infections among fully vaccinated persons, detailed jurisdictional-specific assessments of these cases (e.g., improved understanding of sexual behaviors, such as type of sex and vaccine status of partners) might elucidate risk for infection and potentially guide whether policy about additional vaccine doses should be considered. Regardless of the limitations of this data, the findings from this report indicate that at this time, booster doses are not recommended for patients at risk for mpox exposure during the ongoing outbreak. CDC will continue to monitor these data to assess trends among mpox cases occurring among vaccinated persons so that vaccination guidance can be updated accordingly. Currently, an adequate supply of JYNNEOS vaccine is available; therefore, clinicians can preferentially administer JYNNEOS via the subcutaneous route, although previously administered intradermal vaccine doses were effective and should be considered valid doses and not repeated. Despite concerns about the effectiveness of intradermal doses during the height of the national outbreak, this report reveals that infections among persons who received homologous intradermal doses occurred a median of 100 days later than infections among persons who received homologous subcutaneous doses. The significance of this observation is not known and requires further monitoring and study. 

Clinicians, including those providing care for patients with HIV and other STIs, should counsel patients about the benefits of receiving 2 JYNNEOS vaccine doses to prevent mpox and explain that, although infection can occur among fully vaccinated persons, reports of such infections are rare (less than 1% among the fully vaccinated) and are typically milder than those among unvaccinated persons. Vaccinated persons should employ other prevention strategies in addition to vaccination. Currently available data should support vaccine confidence and encourage mpox vaccination according to the ACIP routine immunization schedule. Persons recommended to receive the vaccine and who received the first dose >28 days ago should receive their second vaccination as soon as possible to complete the 2-dose schedule; ensuring more persons are fully vaccinated will provide better overall protection for individual persons and for communities.

## Footnotes

*
https://www.fda.gov/media/131078/download?attachment

†
https://www.cdc.gov/poxvirus/mpox/response/2022/index.html

§ Data were available through January 10, 2024. Fully vaccinated mpox cases were subsetted to match this same time frame (May 2022–January 2024) to estimate the proportion of infections among all vaccinated cases more accurately. https://www.cdc.gov/poxvirus/mpox/cases-data/mpx-jynneos-vaccine-coverage.html (Accessed March 20, 2024).

¶
https://www.cdc.gov/vaccines/covid-19/downloads/sovc-mpox-052223.pdf

** https://www.cdc.gov/poxvirus/mpox/clinicians/case-definition.html (Accessed March 20, 2024).

††
https://cdn.ymaws.com/www.cste.org/resource/resmgr/ps/ps2022/22-ID-10_Monkeypox_7.28.2022.pdf

§§ Vaccination status was determined by asking whether a patient had been vaccinated against smallpox or monkeypox virus. Dates that each dose was administered were also recorded.

¶¶ Symptom onset date was not always available. Therefore, the estimated illness onset date was assessed using one of the following three possible dates: 1) symptom onset date (when available), 2) date of the most recent positive orthopoxvirus test, or 3) date the surveillance record was created.

*** Data on vaccination coverage were available through January 10, 2024. Fully vaccinated mpox cases were subsetted to match this same time frame (May 2022–January 2024) to estimate the proportion of infections among all vaccinated cases more accurately.

††† 45 C.F.R. part 46.102(l)(2), 21 C.F.R. part 56; 42 U.S.C. Sect. 241(d); 5 U.S.C. Sect. 552a; 44 U.S.C. Sect. 3501 et seq.

§§§ On August 9, 2022, to facilitate access to vaccination and conserve vaccine supply in light of the growing 2022 outbreak, the Food and Drug Administration issued an Emergency Use Authorization to allow for intradermal injection as a 2-dose series (0.1 mL per dose, administered 4 weeks apart). https://www.fda.gov/media/160774/download

¶¶¶ These 31 jurisdictions represented approximately 33% of mpox cases nationally.

**** https://www.cdc.gov/vaccines/schedules/index.html (Accessed April 15, 2024).

†††† https://www.cdc.gov/poxvirus/mpox/prevention/sexual-health.html (Accessed May 17, 2024).
